# Characterization of urinary concentrations of heavy metals among socioeconomically disadvantaged black pregnant women

**DOI:** 10.1007/s10661-020-8163-z

**Published:** 2020-02-27

**Authors:** Inkyu Han, Kristina W. Whitworth, Xueying Zhang, Masoud Afshar, Pamela D. Berens, Elaine Symanski

**Affiliations:** 1 Department of Epidemiology, Human Genetics, and Environmental Sciences, UTHealth School of Public Health, Houston, TX 77030 USA; 2 Southwest Center for Occupational and Environmental Health, Houston, TX 77030 USA; 3 Department of Epidemiology, Human Genetics, and Environmental Sciences, UTHealth School of Public Health in San Antonio, San Antonio, TX 77829 USA; 4 Department of Obstetrics, Gynecology, and Reproductive Sciences, UTHealth McGovern Medical School, Houston, TX 77030 USA; 5 Center for Precision Environmental Health and Department of Medicine, Baylor College of Medicine, Houston, TX 77030 USA

**Keywords:** Socioeconomically disadvantaged, Pregnant women, Urine, Metal exposure, Cotinine

## Abstract

The objective of this study was to characterize exposures to metals using biological samples collected on socioeconomically disadvantaged black pregnant women. We obtained 131 anonymous urine samples provided by black pregnant women visiting a Medicaid-serving prenatal clinic in Houston, TX, from March 27, 2017 to April 11, 2017. We analyzed urine samples for 15 metals including cadmium (Cd), arsenic (As), lead (Pb), and nickel (Ni) and for creatinine and cotinine. We found that median concentrations of zinc (Zn), selenium (Se), and aluminum (Al) among black pregnant women in this study were 1.5 to 3 times higher than levels reported among a cohort of well-educated non-Hispanic white pregnancy planners. We also observed elevated levels of urinary Cd and antimony (Sb) as compared with those reported for a nationally representative sample of adult women in the USA. Based on the results of an exploratory factor analysis, potential sources of metal exposures in this population may arise in home environments or be due to diet, industrial and natural sources, or traffic.

## Introduction

Metals are ubiquitous in the environment. Human exposure to metals may occur through multiple pathways including smoking, inhalation of contaminated ambient air from industrial or vehicular emissions, personal products, and consumption of contaminated food or water (Adamiec et al. 2016; Lough et al. 2005; Samara and Voutsa 2005; Zereini et al. 2005). Compared with organic compounds, many metals are relatively stable and persistent in human tissues and the environment. For instance, cadmium (Cd) is slowly excreted through urine with a urinary half-life close to 40 days (Hu 2000; Wu et al. 2016). Prenatal exposure to environmental chemicals, including metals, is a particular public health concern given the susceptibility of pregnant women and the fetus to the toxic effects of these exposures. Maternal exposure to metals has been associated with increased risk of adverse pregnancy outcomes including preterm birth and low birth weight (Quansah et al. 2015; Bellinger 2005).

Many environmental hazards are unevenly distributed throughout a community such that neighborhoods of color and low socioeconomic status experience higher burden of exposure (Han et al. 2017; Miranda et al. 2011; Pope et al. 2016; Woodruff et al. 2003). Disadvantaged areas are often proximal to hazardous industrial emissions, heavily trafficked roadways, and neighborhood waste disposal sites (Evans and Kantrowitz 2002; Ponce et al. 2005; Linder et al. 2008). Racial disparities in residential soil concentrations of metals have been observed near homes of low-income pregnant women in South Carolina, with higher soil arsenic (As) and lead (Pb) concentrations modeled near homes of black women as compared with homes of white women (Davis et al. 2016). Also, Bell and Ebisu (2012) found that black individuals have higher exposure to particulate matter with aerodynamic diameter smaller than 2.5 μm (PM_2.5_) and to specific PM_2.5_ constituents, including nickel (Ni).

Few studies have characterized biomarkers of metal exposure among socioeconomically disadvantaged pregnant women who likely experience disparities in both environmental exposures as well as in perinatal health outcomes. To our knowledge, only two previous studies have examined urinary metal concentrations among vulnerable populations of pregnant women in the USA, albeit with a focus on mercury (Bashore et al. 2014; Geer et al. 2012). Urinary metal concentrations among pregnant or reproductive-aged women have been described in three other US-based cohorts, though these studies include primarily white women with relatively high socioeconomic status (Davis et al. 2015; Bloom et al. 2015).

We previously published pilot data demonstrating elevated blood concentrations of several metals among 22 pregnant women attending a Medicaid-serving prenatal clinic in Houston, TX (Whitworth et al. 2017). Given the vulnerability and legacy of environmental injustice among black Houstonians in particular, we were interested in building upon our previous study with a focus on black women. Thus, the present study was designed to build upon our previous work by characterizing urinary metal concentrations among socioeconomically disadvantaged black women seeking prenatal care at a large Medicaid-serving prenatal clinic in Houston, TX. We were especially interested in Cd, As, Pb, and Ni, given their potential toxicity (Rebelo and Caldas 2016; Tinkov et al. 2017; Badaloni et al. 2017), previous associations with adverse pregnancy outcomes (Davis et al. 2015; Claus Henn et al. 2016; Fei et al. 2013; Johnston et al. 2014; Llanos and Ronco 2009; Luo et al. 2017; Romano et al. 2016), and our earlier findings (Whitworth et al. 2017). Houston is the fourth largest city in the USA and characterized by a large network of heavily trafficked roadways and dense (e.g., petrochemical plants) and disperse (e.g., metal recycling facilities) industrial sites, presenting ample opportunity for environmental metals exposure. Thus, the objective of this study was to assess concentrations and composition profiles of metals in urine in socioeconomically disadvantaged black pregnant women in Houston.

## Materials and methods

### Study population and sample collection

We obtained a convenience sample of urine specimens (spot urine voids) from black women attending a single prenatal clinic in Houston, TX. This clinic is part of a large university hospital system, located in the Texas Medical Center and serves approximately 16,000 Medicaid or Medicaid-eligible patients annually from across the greater Houston area. This is the same clinic from which women from our previous study (Whitworth et al. 2017) were recruited; however, only black pregnant women were eligible for the current study given our specific interest in this population. All pregnant women attending the prenatal clinic are asked to provide a urine specimen at each prenatal visit as part of standard of care, and the clinic does not often use the entire volume of urine specimen provided by each woman. Thus, we chose to collect urine specimens in this study as an efficient means of obtaining a relatively large number of samples over a short period of time. Over the course of approximately 2.5 weeks in June 2017, nurses in the clinic were asked to set aside urine specimens from black patients (identified from the medical record); during this time period, we obtained 131 anonymous urine specimens. Urine specimens were collected in an ultra-pure grade urine collection cup and placed in a cooler with all identifying information removed. At the end of each day, a field investigator transported specimens from the prenatal clinic to the exposure assessment laboratory at The University of Texas Health Science Center at Houston (UTHealth) School of Public Health, with a transport time of approximately 20 min. Once at the exposure assessment laboratory, specimens were stored in a freezer at − 20 °C until analyses. All collected urine samples were analyzed for metals, creatinine, and cotinine within 2 weeks of sample collection. The study protocol was approved by the UTHealth Institutional Review Board.

### Chemicals and standards

We used inductively coupled plasma mass spectrometry (ICP-MS) calibration standard solutions, internal standard, tuning solutions, and interference check standard solutions (AccuStandard, Inc., New Haven, CT). We quantified 15 metals in urine specimens: silver (Ag), aluminum (Al), As, barium (Ba), Cd, cobalt (Co), chromium (Cr), copper (Cu), Ni, Pb, antimony (Sb), selenium (Se), thallium (Tl), vanadium (V), and zinc (Zn). For internal standards, we used ICP-MS Internal standard solution: scandium (Sc), rhodium (Rh), terbium (Tb), and bismuth (Bi) (AccuStandard, Inc., New Haven, CT). We used trace-metal grade concentrated nitric acid (67% HNO_3_, *w*/*w*, Thermo Fisher Scientific, Waltham, MA).

For creatinine analysis, we used a urinary creatinine colorimetric assay kit (Cayman Chemical, Ann Arbor, MI). The assay kit provided creatinine standard stock solution, creatinine color reagent (1.2% picric acid), 1 M of sodium hydroxide (NaOH), creatinine acid solution (mixture of sulfuric and acetic acid), creatinine sodium borate, and creatinine surfactant solutions.

For cotinine analysis, we used chemicals from Sigma-Aldrich (Sigma-Aldrich, St. Louis, MO): cotinine standard solution with the concentration of 1 mg/mL in methanol, certified reference material (CRM); diphenylamine as an internal standard with the concentration of 5000 μg/mL in methanol, CRM; sodium hydroxide (NaOH), ACS grade ≥ 97%; dichloromethane ≥ 99.8%; and hexane for LC and GC grade (≥ 97%).

### Chemical analysis

On the day of sample analysis, we thawed the urine samples at room temperature and prepared each sample for analyses of 15 metals, creatinine, and cotinine. For metal analysis, we transferred a 0.5-mL urine sample with a disposable plastic pipette into an ultra-pure grade vessel and added 0.5 mL of internal standards (at 1 ppm) consisting of Sc, Rh, Tb, and Bi. We diluted the sample with a mixture of ultra-pure grade nitric acid to make a 2–3% nitric acid with the final volume of 10 mL (1:20 ratio dilution). After the acidification, each tube was centrifuged at 4000 rpm for 15 min using an Eppendorf 5810 (Eppendorf, Hauppauge, NY, USA), and an aliquot from the supernatant was filtered through a Whatman 0.45-μm filter disc. The filtered solution was then introduced into ICP-MS 7500 (Agilent Technologies, Palo Alto, CA) for the analysis of metals. We constructed standard calibration curves ranging from 0.1 to 1000 ng/mL. For each metal, we determined the limit of detection (LOD) as three times the standard deviation of seven replicate analyses of the lowest standard solution (0.1 ng/mL). Thus, the LODs for the 15 metal ranged from 0.14 to 0.66 ng/mL. We also measured metal concentrations in empty urine containers with the same nitric acid solution to assess the background concentrations of metals, which were subtracted from the measured metal concentrations in urine samples. For recovery, we spiked 10 μL of 10 ppm of standard solutions into 10 urine samples. The average recovery rates for all metals were between 106% and 161%. We also conducted replicate analysis in every 15 samples to assess precision. The relative standard deviation in replicate measurements ranged from 3.8 to 13.2%.

For creatinine analysis, we followed the standard operating procedure provided by the manufacturer (Cayman Chemical, Ann Arbor, MI). The detailed procedure has been described previously and is briefly discussed here. We diluted urine samples with HPLC grade deionized water (1:20 dilution). We made standard calibration solutions ranging from 0 to 15 mg/dL from standard stock solution. Following the protocol, we transferred 15 μL of each standard solution using a micropipette into a microcuvette. We also transferred 15 μL of each diluted urine sample using a micropipette into a separate microcuvette. Then, we added 150 μL of alkaline picrate solution to each microcuvette. We incubated all samples at room temperature for 10 min. After the incubation, we placed the microcuvettes into Genesys 10s Spectrophotometer (Thermo Fisher Scientific, Waltham, MA) at the wavelength of 500 nm for the creatinine analysis. We analyzed each sample in duplicate for quality control and assurance. We also reported metal concentrations as μg/g creatinine.

For cotinine analysis, we transferred a 0.5 mL of urine into a 15 mm (inner diameter (ID)) × 100 mm depth glass tube. We added 100 μL of 0.2 ppm diphenylamine as an internal standard. We then added 100 μL of 5 M NaOH solution into the glass tube. We vortexed the glass tube to mix the solutions at 2800 rpm for at least 30 s. After mixing, we added 2.5 mL of dichloromethane-hexane (1:1 *v*/*v*) for liquid-liquid extraction. We used a mechanical liquid-liquid extraction process with vortexing at least 2 min to extract cotinine from urine to the mixture of dichloromethane-hexane. After the liquid-liquid extraction, we transferred the organic layers into another glass tube and centrifuged at 3500 rpm for 3 min. After centrifugation, we transferred the aliquots into K-D concentrator and evaporated the organic solvent until dry at 35 °C with gentle blow of pure nitrogen. We reconstituted the dried samples with 100 μL of hexane and transferred into a 2-mL amber GC/MS vial with a glass insert for the analysis of cotinine. Final aliquots were analyzed for urinary cotinine using GC6890/MSD5973 (Agilent Technologies, Palo Alto, CA). To improve the detection of cotinine, we used programmed temperature vaporizing (PTV) injection using a 50-μL microsyringe with the injection volume of 30 μL (10 μL × 3 injections). The initial inlet temperature was 40 °C, and initial time was 0.45 min. The inlet ramps were as follows: 800 °C/min to 90 °C with 0.5-min hold and 800 °C/min to 320 °C. We set the oven temperature program starting with 40 °C (holding for 4 min), increased to 100 °C with 60 °C/min (no holding), and reached to 320 °C with the ramp of 8 °C/min (hold for 2 min). We used Restek Rxi-5ms column (30-m length × 0.25-mm ID × 0.25-μm film thickness) and pure helium gas as a carrier gas with a flow rate of 1 mL/min. We programmed MS source at 230 °C and MS Quad at 150 °C during the analysis. We simultaneously used scan and selective ion mode (SIM) mode for the quantification of cotinine. The range of scan mode was from 35 to 350 Da. The parameters of SIM mode included group 1 masses (168 and 169) and group 2 masses (98 and 179). We constructed the calibration curve ranging from 0, 50, 100, 200, 500, and 1000 ng/mL. We determined the limit of detection (LOD) as three times the standard deviation of seven replicate analyses of the blank solution. The reported LOD for cotinine was 5.7 ng/mL.

### Data analysis

Observations for which the metal concentration was not detected were assigned a value equal to the LOD divided by the square root of two (Hornung and Reed 1990). All analyses were conducted using SAS version 9.3 (Cary, NC). We classified women as non-smokers if they had a urinary cotinine concentration < 100 ng/mL and as active or passive smokers if their urinary cotinine concentration was ≥ 100 ng/mL (Kim 2016) in order to describe the distribution of urinary metal concentrations stratified by smoking status. We examined the data distributions of urinary metal concentrations and creatinine-adjusted metal concentrations before statistical analysis. We calculated means, standard deviations (SD), medians, and interquartile ranges (IQRs) of urinary metal, creatinine, and cotinine concentrations. We also conducted descriptive statistics using log-transformed data for both unadjusted and creatinine-adjusted metal concentrations. We conducted an exploratory factor analysis among non-smoking women to investigate covariation between log-transformed creatinine-adjusted metal concentrations and to inform potential sources of metal exposure in this population. Due to the different orders of magnitude among metal concentrations, we normalized creatinine-adjusted metal concentrations to standard deviation of 1 and mean of 0 before the factor analysis. The factor analysis was conducted using PROC FACTOR in SAS, using the default factoring method of principal components. Orthogonal rotation, using the VARIMAX option, was performed to retain uncorrelated factors and aid interpretation of factor scores. Based on visual inspection of the scree plot and eigenvalues, we retained three factors, each with an eigenvalue greater than 1.00.

## Results

### Descriptive analyses

We analyzed urinary As concentrations and cotinine in all 131 samples. All other metals were analyzed in 126 samples, and creatinine was analyzed in 125 samples, due to insufficient urine volume. Six metals (Al, As, Ba, Cr, Se, and Zn) were detected in 100% of urine samples (Table 1). For the other nine metals, the proportion of samples with non-detectable concentrations ranged from 2.4% (Cu) to 27.8% (V). Table 1 also presents selected percentiles for the distribution of urinary metal concentrations (μg/L).

**Table 1:** Concentrations (μg/L) of metals in urine specimens from pregnant black women attending a Medicaid-serving prenatal clinic, Houston, TX, 2017Element

	Recovery rate (%)	Precision (%)	LOD (μg/L)	*N* of ND (%)	GM	Mean	SD	Min	25th percentile	50th percentile	75th percentile	Max
Ag	113	8.4	0.142	33 (26.2)	0.25	0.32	0.23	< 0.10	0.10	0.24	0.64	0.91
Al	161	10.7	0.660	0 (0.0)	23.33	45.07	120.70	2.12	8.89	33.39	48.98	1346.64
As	116	4.9	0.282	0 (0.0)	9.52	24.50	40.10	0.21	3.37	5.89	26.10	275.13
Ba	124	3.8	0.170	0 (0.0)	2.77	3.95	3.54	0.27	1.50	3.01	5.26	22.36
Cd	112	10.8	0.176	12 (9.5)	0.37	0.58	0.52	< 0.12	0.14	0.32	1.14	1.86
Co	108	5.8	0.286	36 (28.6)	0.28	0.43	0.50	< 0.20	0.20	0.20	0.44	3.62
Cr	106	5.1	0.303	0 (0.0)	1.50	5.01	5.16	0.21	0.21	5.74	8.62	26.07
Cu	126	13.2	0.316	3 (2.4)	8.00	13.38	14.63	< 0.22	4.52	8.00	17.66	88.80
Ni	107	6.4	0.279	33 (26.2)	0.45	0.81	0.93	< 0.20	0.20	0.26	1.25	4.74
Pb	125	6.1	0.167	33 (26.2)	0.24	0.36	0.47	< 0.12	0.12	0.22	0.45	4.29
Sb	116	6.8	0.154	33 (26.2)	0.29	0.39	0.30	< 0.11	0.11	0.26	0.76	1.27
Se	115	5.5	0.124	0 (0.0)	75.69	101.75	76.87	6.41	50.28	84.31	131.52	498.75
Tl	117	5.0	0.160	33 (26.2)	0.12	0.15	0.10	< 0.11	0.11	0.11	0.19	0.63
V	111	4.9	0.284	35 (27.8)	0.24	0.29	0.24	< 0.20	0.20	0.20	0.26	2.40
Zn	118	6.5	0.361	0 (0.0)	402.53	559.78	504.80	45.28	231.35	368.10	741.63	2874.52

Total number of samples is 126 for all metals except As (*n* = 131). Precision shown as relative standard deviation (%) among 10 replicate samples

*LOD* limit of detection, *Max* maximum, *Min* minimum, *ND* non-detect, *GM* geometric mean, *SD* standard deviation

Table 2 shows summary statistics for the creatinine-adjusted urinary metal concentrations. The highest concentrations were observed for Zn (GM = 276.17 μg/g creatinine, median = 267.47 μg/g creatinine) followed by Se (GM = 51.85 μg/g creatinine, median = 46.59 μg/g creatinine) and Al (GM = 16.10 μg/g creatinine, median = 17.73 μg/g creatinine). GM concentration of urinary As was 6.25 μg/g creatinine (median = 6.63 μg/g creatinine) whereas the GM concentrations of urinary Cd, Ni, Pb, and Sb were less than 0.31 μg/g creatinine. We found that creatinine-adjusted Ag, Cd, and Sb were highly correlated with each other (Spearman correlation ranging from 0.92 to 0.98, *p* < 0.001). Creatinine-adjusted Al was positively associated with creatinine-adjusted Cr (*r* = 0.87, *p* < 0.001). Creatinine-adjusted V was also highly correlated with creatinine-adjusted Co (*r* = 0.85, *p* < 0.001).

**Table 2:** Creatinine-adjusted concentrations (μg/g) of metals in urine specimens from pregnant black women attending a Medicaid-serving prenatal clinic, Houston, TX, 2017

Element	Number	GM	Mean	SD	Min	25th percentile	50th percentile	75th percentile	Max
Ag	125	0.17	0.34	0.50	0.10	0.09	0.16	0.32	1.46
Al	125	16.10	35.14	59.84	0.68	5.27	17.73	43.72	559.07
As	130	6.25	17.21	25.83	0.06	2.04	6.63	23.90	168.30
Ba	125	1.89	3.43	4.63	0.16	0.95	1.81	3.85	37.10
Cd	125	0.26	0.57	0.96	0.01	0.10	0.21	0.55	7.75
Co	125	0.19	0.34	0.39	0.01	0.09	0.20	0.47	2.21
Cr	125	1.04	4.90	7.06	0.01	0.14	2.22	7.27	48.91
Cu	125	5.50	10.63	16.71	0.32	2.79	5.37	10.81	138.45
Ni	125	0.31	0.63	0.93	0.00	0.14	0.30	0.76	6.76
Pb	125	0.17	0.29	0.46	0.02	0.08	0.13	0.34	3.51
Sb	125	0.20	0.40	0.65	0.02	0.08	0.16	0.44	5.27
Se	125	51.85	80.52	108.55	5.95	26.92	46.59	101.49	943.42
Tl	125	0.08	0.15	0.24	0.01	0.05	0.09	0.16	2.29
V	125	0.17	0.25	0.23	0.01	0.09	0.16	0.32	1.46
Zn	125	276.17	486.59	1088.12	18.25	156.07	267.47	471.91	11,715.34

*GM* geometric mean, *max* maximum, *min* minimum, *SD* standard deviation

### Effects of smoking

**Fig. 1:**
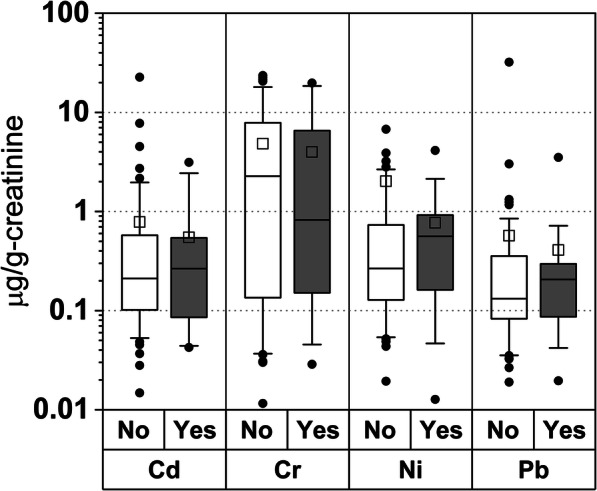
Box plots of selected metals (Cd, Cr, Ni, and Pb) by smoking status during pregnancy among 125 pregnant women. *X*-axis shows four metals with smoking status (No: no smoking (*n* = 104) vs. Yes: active or passive smoking (*n* = 21)). *Y*-axis represents metal concentrations adjusted for creatinine in urine. Whiskers represent outliers, and open squares represent mean values. Horizontal lines in the boxes show median values. Bottoms and tops of the boxes represent 25th and 75th percentiles for each metal

In our study, 14 of 131 samples had cotinine concentrations greater than 200 ng/mL. Seven samples had cotinine concentrations between 106 ng/mL and 188 ng/mL (median = 133 ng/mL). The rest of urine samples with cotinine levels between 9.3 and 94 ng/mL (median = 24 ng/mL). We considered both active smokers (*n* = 14) and passive smokers (*n* = 7) as the exposed group (*n* = 21). We conducted non-parametric Wilcoxon scores rank sum test to compare the differences of each metal between the exposed group (active smokers and passive smokers) and unexposed group (no smokers). We did not find significant differences in the mean values of each of the 15 metals between groups. Figure 1 shows the box plots of creatinine-adjusted Cd, Cr, Ni, and Pb between exposed and non-exposed groups. Median values of Cd, Ni, and Pb were higher for exposed group than unexposed group; however, the differences between two groups were not statistically significant.

### Exploratory factor analysis

Results from our factor analysis are summarized in Table 3. Three factors were identified when only factors with eigenvalues > 1.0 were retained. Factors 1, 2, and 3 accounted for 37, 33, and 30%, of the total variance, respectively. Factor 1 consisted of Ag, Cd, Sb, Tl, and Pb. In factor 2, urinary Al, Cr, V, and Co were included with high factor loadings (> 0.600). Lastly, we found that factor 3 included urinary Ni, Cu, Zn, and Se.

**Table 3:** Results of exploratory factor analysis of urinary metal concentrations among 125 black women attending a Medicaid-serving prenatal clinic in Houston, TX, 2017

Metal	Factor 1	Factor 2	Factor 3
As	0.295	0.291	0.464
Al	0.269	*0.813*	0.259
V	0.189	*0.685*	0.471
Cr	0.199	*0.913*	0.109
Co	− 0.016	*0.685*	0.448
Ni	− 0.087	0.349	*0.711*
Cu	0.045	0.096	*0.825*
Zn	0.243	− 0.418	*0.674*
Se	0.222	0.207	*0.844*
Ag	*0.937*	0.275	0.039
Cd	*0.923*	0.232	0.030
Sb	*0.944*	0.257	0.041
Ba	0.455	0.580	0.264
Tl	*0.624*	0.560	0.262
Pb	*0.743*	− 0.193	0.403
Variance explained (%)	4.12 (37%)	3.75 (33%)	3.37 (30%)
Final communality estimates	11.24		

Italic text represents factor loadings greater than 0.600

## Discussion

This pilot study was conducted to characterize exposures to metals in urine collected from 131 socioeconomically disadvantaged black pregnant women visiting a Medicaid-serving prenatal clinic in Houston, TX. About 16% of study participants (*n* = 21) were exposed to smoking during their pregnancy. However, we did not find significant effects of smoking on metal exposures. We identified three significant factors informing potential exposure sources in the exploratory factor analysis.

We are aware of only one other study describing urinary concentrations of Zn and Se among pregnant women. The GM concentration of Zn in our study (402.5 μg/L) was approximately twice as high as that reported from 380 pregnant women in the LIFECODES cohort study in Boston (median = 203.6 μg/L) (Kim et al. 2019). Similarly, we observed a twofold increase in the GM concentration of urinary Se (75.69 μg/L) as compared with the GM concentration (35.4 μg/L) reported by Kim et al. from the LIFECODES cohort (Kim et al. 2019). It is possible that the differences in results might be affected by different racial/ethnic and socioeconomic composition of study participants. The population in the Kim et al. (2019) consisted of largely well-educated non-Hispanic white women. In contrast, urine specimens in our study were provided by African-American women who sought care from a prenatal clinic that serves primarily Medicaid recipients. The potential impact of prenatal exposure to Zn and Se, which are considered essential elements for humans, on reproductive and perinatal health outcomes is unknown. However, there is evidence that elevated levels of these compounds may be associated with adverse cardiovascular outcomes such as hypertension (Wu et al. 2018; Laclaustra et al. 2009). Kim et al. (2019) also found a positive association between Se concentrations among the pregnant women in their study and urinary concentrations of 8-isoprostane and 8-hydroxydeoxyguanosine (8-OHdG), biomarkers of oxidative stress (Kim et al. 2019).

Two previous studies of pregnant women in the US have reported on urinary concentrations of As. The median urinary As concentrations in our study was 5.89 μg/L, which is higher than was reported by Davis et al. (2015) among primarily white pregnant women in New Hampshire (3.1 μg/L). However, our GM As concentrations (9.52 μg/L) were lower than what was reported among pregnant women in the LIFESTAGES cohort, in Boston (15.0 μg/L). Prenatal exposure to As in drinking water has been previously associated with spontaneous abortion, low birth weight, and still birth, primarily in developing countries, such as Bangladesh, with endemic As contamination (Milton et al. 2017; Mohammed-Abdul et al. 2015). While the association between urinary As and reproductive and perinatal health outcomes among women in the USA has not been fully explored, Davis et al. (2015) did report negative associations between maternal urinary As concentrations (at levels lower than observed in our population) and reduced fetal head circumference.

The GM concentrations of Ni and Pb from urine specimens in the present study were 0.45 μg/L and 0.24 μg/L, respectively, and just over one quarter of the samples were below the LOD. These concentrations were lower than reported in the LIFECODES cohort (Ni GM 2.46 μg/L and Pb GM 0.30 μg/L). Nickel is a toxic compound and classified as a human carcinogen (IARC 2012; NTP 2014); nonetheless, the potential maternal and child health impact of prenatal Ni exposure is not well understood. In a 2015 systematic review, McDermott et al. (2015) identified only six studies evaluating associations of prenatal Ni exposure with multiple perinatal and children’s health outcomes, with varied methodology and mixed results (McDermott et al. 2015). The impact of maternal Pb exposure on maternal and child health outcomes has been relatively well studied, with associations between prenatal Pb exposures and child neurodevelopment as well as with reproductive and perinatal outcomes (Rahman et al. 2003; Bellinger 2005).

Median concentration of urinary Cd in this study (0.32 μg/L) was similar to that reported among women (age of 20 and older) in earlier waves of the National Health and Nutrition Examination Survey (NHANES) (1999–2006) (median = 0.36 μg/L) (Peng et al. 2017) but higher than reported in NHANES from 2007 to 2008 (median = 0.20 μg/L) (Yorita Christensen 2013). Gallagher et al. (2011) evaluated racial/ethnic differences in creatinine-adjusted urinary Cd concentrations among women aged 20–49 years in NHANES 2003–2008 and reported a mean concentration of 0.27 μg/g creatinine among 146 black women (Gallagher et al. 2011), a value lower than found in the present study (creatinine-adjusted Cd concentration among non-smoking black women, mean = 0.57 μg/g creatinine or GM = 0.26 μg/g creatinine). Comparisons between our results and NHANES data among non-pregnant population should be made with caution, however, as pregnancy is a time of immense physiological changes that may impact measured concentrations of chemicals. However, our GM concentration of Cd (0.37 μg/L) was also orders of magnitude larger than observed among pregnant women in the LIFECODES study (0.04 μg/L).

Recently, there is growing evidence that exposure to antimony (Sb) is associated with increased risk for mortality, sleep disorder, and other adverse health outcomes (Guo et al. 2016; Scinicariello et al. 2017; Scinicariello and Buser 2016). In early 2000, median concentrations of urinary Sb ranged from 0.04 to 0.05 μg/L in the USA (Bloom et al. 2015; Yorita Christensen 2013) whereas a recent NHANES study reported geometric mean levels of urinary Sb for males and females of 0.12 μg/L (Scinicariello and Buser 2016). Geometric mean levels of urinary Sb in our study (0.29 μg/L) were higher than those in the aforementioned previous studies. Though again, comparisons with non-pregnant populations should be made with caution; thus, while these previous studies may be an imperfect comparison, to our knowledge, there are no other studies reporting urinary concentrations of Sb among pregnant women in the USA. The potential impact of elevated levels of Sb in urine on adverse health outcomes for pregnant women should be considered in future environmental health studies.

In our study, we classified 16% (*n* = 21) of 131 samples having cotinine concentrations greater than 100 ng/mL as an active or passive smoker (Kim 2016). The rest of samples were categorized as having come from non-smokers. We did not find significant difference of urinary metal concentrations between smoking and non-smoking during pregnancy. For instance, urinary concentrations of Cd in our study were lower for smokers (GM = 0.36 μg/L or GM = 0.24 μg/g creatinine) than non-smokers (GM = 0.38 μg/L or GM = 0.26 μg/g creatinine) although there was no significant difference between them. In contrary, urinary concentrations of Pb in our study were higher for smokers (GM = 0.29 μg/L or GM = 0.19 μg/g creatinine) than non-smokers (GM = 0.23 μg/L or GM = 0.16 μg/g creatinine), and the difference was not significant. However, given the small number of active and passive smokers (*n* = 21), interpretation of these results should be made with caution. In general, these metals in urine voids for smokers were higher than non-smokers. The NHANES conducted between 1999 and 2004 reported urinary concentrations of Cd for female smokers (GM = 0.40 μg/L or GM = 0.37 μg/g creatinine) were significantly higher as compared with female non-smokers (GM = 0.26 μg/L or GM = 0.20 μg/g creatinine). The study also reported that unadjusted and adjusted urinary concentrations of Pb for female smokers were significantly higher than female non-smokers (Richter et al. 2009).

The exploratory factor analysis identified three significant factors, informing potential metal exposure sources in our population. Factor 1 (Ag, Cd, Sb, Tl, and Pb) may be related to sources in the home and personal environments, hobbies and foodstuffs such as the use of ceramics (Sb and Pb), flame retardants (Sb) in clothing and home furnishings (Edelman et al. 2003), jewelry (Ag) (Drake and Hazelwood 2005), and dietary intake of fruits and vegetables (Cd and Tl) (Kazantzis 2000; Jarup et al. 1998). Metals that loaded on Factor 2 (Al, V, Cr, and Co) might be associated with either industrial or natural sources. Although Al, V, and Cr from crustal materials are naturally present in soil, these elements are also emitted from petrochemical plants and refineries, from burning heavy oil for shipping vessels, and from other metal-related industrial facilities (Moreno et al. 2010; Bozlaker et al. 2017; Han et al. 2017), which are abundant in the greater Houston area (US EPA 2019). Factor 3 (Ni, Cu, Zn, and Se) consisted of metals that may be associated with traffic sources such as vehicular and railroad emissions (e.g., break wear) (Harrison et al. 2012; Ha et al. 2014). Factor 3 may be associated with dietary intake of tap water where metals (Ni and Cu) have leached from plumbing pipes or through dietary supplement (Se) (Klaassen 2013). However, the association between these sources and metal exposure is not straight forward without detailed time activity and diet data.

As a pilot study, one limitation of this study was the lack of information about lifestyle and sociodemographic characteristics such as age, education, or residence. However, we measured urinary cotinine to evaluate the likelihood of being a smoker or being exposed to environmental tobacco smoke, two sources of metal exposure. We collected spot urine samples as the participants visited to clinic. Thus, we were not able to obtain representative urine samples during the day, although this may not heavily affect the overall distribution of metals among our study participants (aee creatinine-adjusted summary in Table 2). Because urine specimens were anonymous, we cannot be certain that individual women are only represented once in our dataset. Given the short time from which specimens were collected (approximately 2.5 weeks), it is improbable, though not impossible, that women visited the prenatal clinic more than once during that time period and, thus, contributed more than one urine specimen to our study. Even in the event that this did happen, it is not likely that it occurred for more than a few women.

Despite the limitations in our study, there were several strengths as well. We measured a suite of metals in urine from socioeconomically disadvantaged black pregnant women, a group that is largely underrepresented in the literature. Also, this study was conducted in Houston, TX, the fourth most populous city in the USA and one with a history of environmental injustice and potentially multiple sources of toxic environmental exposures (Chakraborty et al. 2011; Linder et al. 2008; Sexton et al. 2007). In addition to a vast network of heavily trafficked roadways, the Houston Ship Channel, home to one of the world’s largest petrochemical complexes and a busy port, is located on the east side of the city. Moreover, there are more than a dozen Superfund sites in the greater Houston area, several known to be contaminated with toxic metals and many situated along Houston’s complex system of bayous (TCEQ 2017). Despite great opportunity for exposure, to our knowledge, our study represents the first characterization of this population’s exposure. Exposure information about metals during pregnancy provides needed data to understand the overall impact of multiple sources of metals in this population. Additional research is necessary to understand the effects of exposure to metal mixtures on physiological changes to assess adverse health outcomes.

## Conclusion

Among 131 anonymous urine samples provided by socioeconomically disadvantaged black pregnant women, we found that median concentrations of Zn, Se, Al were higher than other metals. Moreover, urinary concentrations of As, Zn, Se, and Cd were elevated in our study participants compared with other studies of pregnant women. Because the literature characterizing urinary metal concentration among pregnant women in the USA is quite small and has been primarily among white women with higher socioeconomic status, the ability to place our results within the context of this previous literature is hindered and further highlights a data gap in understanding metals exposures among overburdened minority populations in the USA.
